# Postoperative pneumonia after femoral fracture surgery: an in-depth retrospective analysis

**DOI:** 10.1186/s12891-024-07529-4

**Published:** 2024-05-27

**Authors:** Mohammad Hamdan, Bassem I. Haddad, Jamil Almohtasib, Mira Eid, Tasneem Jamal Al-Din, Hashem A. Rayyan, Ahmad M. Altantawi, Abdussalam S. Akaheal, Mohammad Ali Alshrouf

**Affiliations:** 1 Department of Special Surgery, Division of Orthopedics, School of Medicine, The University of Jordan, Amman, 11942 Jordan; 2 School of Medicine, The University of Jordan, Amman, 11942 Jordan

**Keywords:** Pneumonia, Femoral fracture, Postoperative complications, Orthopedic procedures, Prevention

## Abstract

**Background:**

Femoral fractures significantly contribute to disability, predominantly in the elderly. Despite this, data on postoperative pneumonia following femoral fracture surgeries remains sparse. Our study sought to explore the incidence and impact of postoperative pneumonia on outcomes following such surgeries.

**Methods:**

A retrospective study analyzed femoral fracture patients hospitalized from 2016 to 2022. We scrutinized postoperative outcomes, including pneumonia, hospital stay duration, intensive care unit (ICU) admissions, and in-hospital mortality. We established stringent diagnostic criteria for postoperative pneumonia, incorporating both clinical signs and radiological evidence, excluding patients with prior infections or those discharged within 24 h post-surgery. Statistical analyses involved Chi-square and t-tests, linear regression, and logestic regression using SPSS.

**Results:**

Out of 636 patients, 10.8% were diagnosed with postoperative pneumonia. The average age was 79.55 ± 8.57 years, with a male prevalence of 47.8%. Common comorbidities were hypertension (78.3%), diabetes (60.9%), and cardiovascular diseases (40.6%). Surgical interventions were categorized as intramedullary nailing (40.6%), partial hip replacement (37.7%), and dynamic hip screw (21.7%). Postoperative pneumonia was associated with older age (AOR = 1.053, 95% CI 1.020 to 1.087, *p* = 0.002), ICU admission (AOR = 2.283, 95% CI 1.256 to 4.148, *p* = 0.007), and longer length of hospital stay (AOR = 1.079, 95% CI 1.030 to 1.130, *p* = 0.001). The presence of pneumonia was associated with a 2.621-day increase in hospitalization after adjusting for other variables (*p* < 0.001, 95% CI: 1.454 to 3.789).

**Conclusion:**

This study accentuates the clinical significance of postoperative pneumonia in femoral fracture patients, with a noted incidence of 10.8%. A notable association with older age, prolonged hospital stays, and ICU admissions was observed, underscoring the necessity of addressing this complication to improve patient outcomes and healthcare resource allocation.

## Introduction

Femoral fractures are recognized as a serious, debilitating problem worldwide, especially concerning the geriatric population. As this issue continues to rise, with an annual estimate of 1.6 million patients with a hip fracture hospitalized [1], the number of hip fracture surgeries simultaneously expands alongside their associated complications, like postoperative pneumonia. It has been estimated that the total annual incidence of geriatric hip fractures in the Middle East in general is between 60 and 150 per 100,000 [2–5]. There is a scarcity of research examining the incidence and complications of hip fractures among the Jordanian population. According to recent research in Jordan, it was estimated that the annual incidence of hip fracture patients above the age of 55 in 2021 was approximately 96 cases per 100,000 individuals [6].

Factors such as advanced age, anemia, diabetes, prior stroke, the number of comorbidities, an American society of anesthesiologists (ASA) score ≥ III, general anesthesia, and delay in surgery were positively correlated to acquiring pneumonia after surgery [7, 8]. On the other hand, many elements, regardless of pneumonia, were found to affect the length-of-stay (LOS) following a hip fracture surgery. These included advanced age, higher ASA physical status scores, comorbid burden, with the addition of female gender, severe obesity with a body mass index (BMI) exceeding 40, the use of a cemented implant in the total hip replacement, previous hip fractures, acute renal failure, diabetes, cerebrovascular disease, smokers, and others [9–11]. Others were linked with death after these surgeries. For instance, longer LOS, age over 80, poor mobility prior to the surgery, inability to return to baseline mobility, the presence of 3 or more comorbidities, an ASA over III or IV, chest infection, and heart failure [10, 12, 13]. In the management of hip fractures, particularly among elderly patients, postoperative complications significantly influence outcomes and mortality rates [14–16]. Among these complications, pneumonia stands out as a critical risk factor. The incidence of pneumonia following surgery not only complicates recovery but also markedly increases the risk of mortality [14, 16, 17]. This relationship is particularly pronounced in geriatric patients, who may already present with compromised health status pre-surgery.

While many of the aforementioned factors overlap, very limited data is available regarding the complications associated with the development of postoperative pneumonia after femoral fracture surgeries. Some evidence suggests that postoperative pneumonia significantly increased the 30-day mortality to be 27–43%, prolonged the hospital stay by 56%, increased the rate of sepsis by about 10%, and increased the risk of readmission by eightfold [18, 19].The exact relationship between the incidence of pneumonia and these numerous complications is not well understood, and there are variable trends in this regard, which calls for further investigation. Moreover, studies suggest that the 30-day mortality was higher in hip fracture patients with coronavirus disease (COVID-19) infection; however, vaccinated patients with COVID-19 infection had a comparable mortality risk to those without the virus, indicating that the illness was less severe [20, 21].

The incidence and implications of postoperative pneumonia have been a worthy topic of discussion, and scholars have attempted to investigate the potential risk factors for postoperative pneumonia following surgically treated femoral fractures in elderly patients [7, 18, 19, 22, 23]. Due to the lack of such data in this population, this study aims to determine the incidence and effects of postoperative pneumonia after femoral fracture surgery on the length-of-stay in the hospital, as well as the mortality rate and factors associated with postoperative pneumonia in the hospital in the Jordanian population. We hypothesize that the effects of pneumonia on our sample would increase both factors involved. Therefore, the results emerging from this study are expected to help optimize the care provided to these patients and eventually improve their quality of life.

## Methodology

### Study design

We conducted a retrospective cohort study using data that was prospectively collected from patients diagnosed with femur fractures who have undergone surgical treatment at Jordan University Hospital between the years 2016 and 2022. Prior to the study’s beginning, the protocol was evaluated and approved by the Jordan University Hospital ethics committee, and the appropriate institutional review board (IRB) approved the study proposal (approval number 101,202,315,854; 2/3/2023). The Code of Ethics of the World Medical Association (Declaration of Helsinki) was followed while conducting the study.

### Study population

The research includes patients diagnosed with femoral fractures, confirmed through imaging, and admitted to the University of Jordan Hospital within the time frame of 2016 to 2022. There are no specific limitations regarding the time frame between the occurrence of the fracture and admission. Inclusion criteria cover patients who underwent femoral fracture surgery as confirmed through imaging (e.g., X-rays, computed tomography (CT) scans), patients admitted to the University of Jordan Hospital within the specified time frame of 2016 to 2022, and had complete medical records for analysis. Exclusion criteria involve patients discharged within 24 h of admission, to ensure that we could accurately capture cases of postoperative pneumonia, which typically do not manifest immediately after surgery, and patients who were diagnosed with other infectious diseases (such as respiratory infections and urinary tract infections). The diagnosis was based on the presence of any symptoms or signs and additional diagnostic tests that were necessary, and this was done to avoid any possible confounding effects and focus only on the incidence and effects of postoperative pneumonia related to the surgery.

### Data collection

The data were systematically retrieved from electronic medical records at Jordan University Hospital. The data encompassed vital patient information, such as gender and age. A comprehensive evaluation of previous medical history was done to identify conditions like diabetes, hypertension, and malignancies, along with any other cardiovascular, pulmonary, renal, and neurological diseases. Pulmonary co-morbidity was based on the patient having one of the following: asthma, COPD, or pulmonary fibrosis. Factors related to surgery, including if the surgery was performed within 48 h, type of fracture (classified according to the International Classification of Diseases, Tenth Revision (ICD-10) codes). These included femur neck fractures (S72.0), pertrochanteric fracture (S72.1), intertrochanteric fractures (S72.14), subtrochanteric fractures (S72.2), and femoral shaft fractures (S72.3)), type of surgical procedure (dynamic hip screw (DHS), intramedullary nailing (IMN), partial hip replacement (PHR), proximal femoral nail antirotation (PFNA)), and type of anesthesia (general vs. spinal) were also scrutinized. The hemoglobin level upon admission was also collected as a standard procedure aimed at evaluating patients’ overall health status and identifying potential risks associated with surgery, such as anemia or other hematological conditions. Additionally, the study took into account post-operative outcomes by assessing the occurrence of post-operative pneumonia, length of hospital-stay, and in-hospital mortality. In our study, strict hospital guidelines were implemented to control COVID-19 infections among patients. These guidelines mandate that all patients undergo a COVID-19 polymerase chain reaction (PCR) test within 48 h prior to the operation, and positive cases were quarantined and the surgery delayed.

### Diagnosis of pneumonia

Post-operative pneumonia was diagnosed by the respiratory team in the hospital, depending on the clinical findings of the patient and an examination of the chest X-ray findings after surgery. The assessment of chest X-ray involved an examination by experienced radiologists, with specific attention to the presence of infiltrates, consolidations, or other abnormalities indicative of pneumonia. In addition, the clinical assessment included a thorough examination, which included the evaluation of temperature, respiration rate, white blood cell count, chest physical examination, and other relevant indicators.

### Statistical analysis

All collected data was cleaned, coded, and analyzed on SPSS version 27. Categorical variables (e.g., gender) were presented as frequencies n (%), while continuous variables (e.g., age) were presented as means ± standard deviations. Mean differences in responses and domain scores were examined using the independent t-test and Chi-square test. Femoral fracture patients were split according to their diagnosis of pneumonia; mean differences were examined using the independent t-test. Variables that showed univariate analysis with a *p* < 0.1 were included in the logistic regression model in order to control for possible confounding factors for the predictors of post-operative pneumonia, which were summarized using adjusted odds ratio (AOR). A linear regression model was conducted to test the effect of post-operative pneumonia on the length of stay, adjusted for variables with *p* < 0.1 in univariant analysis. All statistical tests are conducted with a 95% confidence interval and a 5% error margin. A p-value of less than 0.05 is considered statistically significant.

## Results

A total of 636 patients were included in our study between 2016 and 2022. Of which, 69 patients (10.8%) were treated for post-operative pneumonia. The mean age for these patients was 79.55 ± 8.57, with 47.8% being male. Hypertension (78.3%), diabetes (60.9%), and cardiovascular disease (40.6%) were the most prevalent co-morbidities. Table 1 demonstrates the patient characteristics and clinical information in relation to the diagnosis of pneumonia.

**Table 1:** Patient characteristics and clinical information in relation to the diagnosis of pneumonia

	Total (*n* = 636)	Diagnosis of pneumonia		Crude OR	*P*-value
		Yes (*n* = 69)	No (*n* = 567)		
**Age**	75.82 ± 10.92	79.55 ± 8.57	75.37 ± 11.09	-	**0.003** ^**a**^
**Male**	304 (47.8%)	35 (50.7%)	269 (47.4%)	1.140 (0.692–1.880)	0.606^b^
**History of**:					
Hypertension	431 (67.8%)	54 (78.3%)	377 (66.5%)	1.814 (0.998–3.299)	**0.048** ^**b**^
Diabetes	314 (49.4%)	42 (60.9%)	272 (48.0%)	1.687 (1.012–2.812)	**0.043** ^**b**^
CVD	185 (29.1%)	28 (40.6%)	157 (27.7%)	1.783 (1.066–2.983)	**0.026** ^**b**^
Renal disease	89 (14.0%)	14 (20.3%)	75 (13.2%)	1.670 (0.885–3.151)	0.110^b^
CVA	75 (11.8%)	10 (14.5%)	65 (11.5%)	1.309 (0.638–2.685)	0.461^b^
Malignancy	45 (7.1%)	6 (8.7%)	39 (6.9%)	1.289 (0.525–3.166)	0.578^b^
Pulmonary	37 (5.8%)	6 (8.7%)	31 (5.5%)	1.647 (0.661-4.100)	0.279^b^
Dementia	36 (5.7%)	4 (5.8%)	32 (5.6%)	1.029 (0.353–3.002)	0.958^b^
Neuro disease	18 (2.8%)	2 (2.9%)	16 (2.8%)	1.028 (0.231–4.569)	0.971^b^
**HB level on admission**	11.91 ± 2.38	11.75 ± 2.31	11.93 ± 2.39	-	0.559^a^
**Type of surgery**					
IMN	269 (42.3%)	28 (40.6%)	241 (42.5%)	ref	
PHR	211 (33.2%)	26 (37.7%)	185 (32.6%)	1.210 (0.686–2.133)	0.511
DHS	156 (24.5%)	15 (21.7%)	141 (24.9%)	0.916 (0.473–1.773)	0.794
**Type of anesthesia**					
Spinal	432 (67.9%)	47 (68.1%)	385 (67.9%)	ref	
General Anesthesia	204 (32.1%)	22 (31.9%)	182 (32.1%)	0.990 (0.579–1.693)	0.971
**Surgery done within 48 h (yes)**	502 (78.9%)	44 (63.8%)	458 (80.7%)	0.419 (0.246–0.714)	**0.001** ^**b**^
**ICU admission (yes)**	168 (26.4%)	37 (53.6%)	131 (23.1%)	3.848 (2.307–6.420)	**< 0.001** ^**b**^
**Mortality (yes)**	7 (1.1%)	2 (2.9%)	5 (0.9%)	3.355 (0.638–17.633)	0.130^b^
**Length of hospital stay**	8.07 ± 5.24	12.04 ± 8.43	7.59 ± 4.5	-	**< 0.001** ^**a**^

*Note*^a^Independent t-test, ^b^Chi-square; OR, odds ratio; CVD, cardiovascular disease; CVA, cerebrovascular accident; Hb, hemoglobin; IMN, intramedullary nailing; DHS, dynamic hip screw; PHR, partial hip replacement; ICU, intensive care unite

In a logistic regression to investigate the influence of postoperative pneumonia on variables with a p-value less than 0.1, the results revealed positive statistically significant predictors including older age, length of stay, and ICU admission. Table 2 showcases the outcomes of the regression model, examining the influence of pneumonia on various clinical parameters.

**Table 2:** Regression model for pneumonia’s impact on clinical outcomes

Variables	Postoperative pneumonia			
	Adjusted OR	Lower Bound	Upper Bound	*P*-value
Age	1.053	1.020	1.087	**0.002**
Hypertension	1.348	0.679	2.677	0.394
Diabetes mellitus	1.478	0.813	2.686	0.200
Cardiovascular disease	1.229	0.686	2.201	0.488
Surgery done within 48 h	0.806	0.423	1.537	0.513
ICU admission	2.283	1.256	4.148	**0.007**
Length of hospital stay	1.079	1.030	1.130	**0.001**

*Note* OR, odds ratio; ICU, intensive care unite

**Fig. 1:**
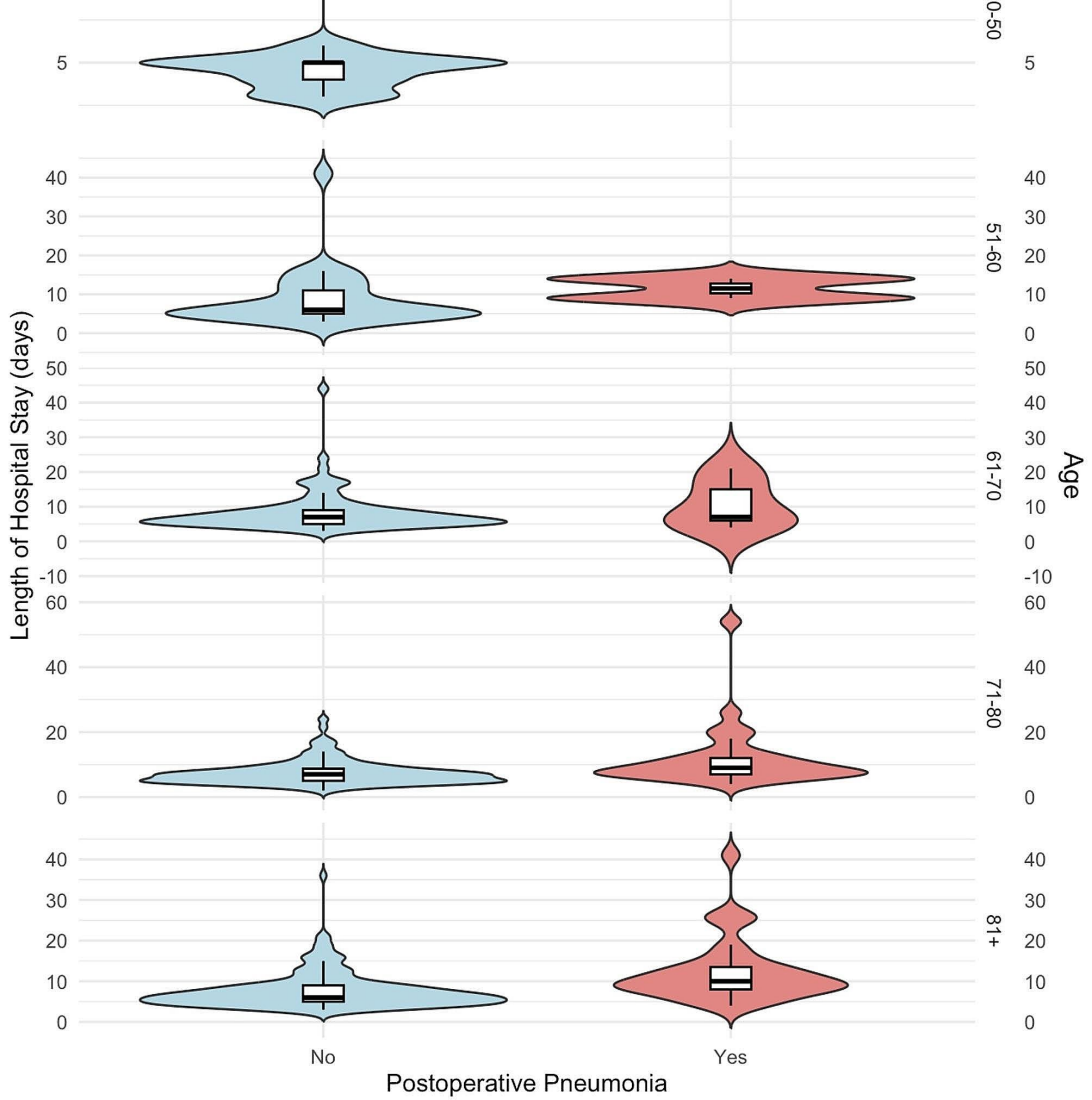
Boxplot on top of the violin plot to demonstrate the impact of postoperative pneumonia on length of hospital stay with a subgroup analysis by age groups

We used linear regression to analyze the predictors of length of hospital stay. Relevant variables with a p-value < 0.1 in univariate analysis were entered into the model. Our analysis revealed that post-operative pneumonia and ICU admission were positively associated with the length of hospital stay, indicating longer stays. Conversely, higher hemoglobin levels and surgery performed within 48 h of admission were negatively associated with the length of hospital stay, indicating shorter stays (Table 3). Figure 1 represents a boxplot overlaid on top of a violin plot, illustrating the influence of postoperative pneumonia on the length of hospital stay, stratified by age groups. It demonstrates a significant increase in the length of hospital stay for patients diagnosed with pneumonia within the age groups of 71–80 and + 81 years (*p* < 0.05).

**Table 3:** Regression analysis results for factors influencing length of hospital stay

Variables	Length of hospital stay			
	β	Lower Bound	Upper Bound	*P*-value
Hypertension	0.108	-0.656	0.872	0.782
Cardiovascular disease	0.429	-0.376	1.235	0.296
Hemoglobin level	-0.152	-0.300	-0.005	**0.042**
Surgery done within 48 h, yes	-3.756	-4.659	-2.853	**< 0.001**
Postoperative pneumonia diagnosis, yes	2.621	1.454	3.789	**< 0.001**
ICU admission	3.498	2.658	4.338	**< 0.001**

*Note*, beta coefficient for linear regression; ICU, intensive care unite

## Discussion

Our retrospective study, which included 636 patients who had surgical procedures for femoral fractures from 2016 to 2022, provides insights into the prevalence and outcomes of postoperative pneumonia in this specific population. Significantly, 10.8% of patients were diagnosed with post-operative pneumonia. The participants had a mean age of 79.55 ± 8.57 and a balanced male-to-female ratio. Postoperative pneumonia was associated with older age, ICU admission, and a longer length of hospital stay. The presence of pneumonia was associated with a 2.621-day increase in hospitalization after adjusting for other variables. In addition, prolonged hospital stay was associated with surgery not being performed within 48 h of admission, lower hemoglobin levels upon admission, and ICU admission.

The effect of postoperative pneumonia (POP) on the duration of hospitalization following femoral fracture surgeries is a crucial aspect to consider when assessing patient care. Consistent with the existing literature, our study revealed that patients diagnosed with POP experience a longer hospital stay and are more likely to be admitted to the ICU [1]. This extended stay not only bears repercussions for individual patients but also exerts an effect on healthcare institutions by increasing the cost of care and utilizing scarce resources inefficiently. Therefore, it is essential to minimize the risk of POP by identifying high-risk patients and implementing strategies for the early detection and treatment of pneumonia. In a cohort study designed to evaluate a standardized POP prevention program, Kazaure et al. showcased a notable decrease of 43.6% in POP rates post-implementation [24]. Additionally, in a non-randomized, quasi-experimental study, Chang et al. highlighted the benefits of pulmonary rehabilitation in elderly patients with hip fractures, demonstrating a significantly lower incidence of POP and shorter hospital stay in patients receiving chest physiotherapy on the first post-surgery day in comparison to the control group [25]. Therefore, a multifaceted approach focused on identifying high-risk patients and adopting comprehensive preventive and management strategies emerges as a critical factor in mitigating the risk of POP and subsequently reducing the duration of hospitalization and ICU admissions.

On the other hand, the lack of a significant relationship between mortality and pneumonia in our population warrants further investigation and exploration, as it contradicts previous literature [19]. Jang et al. studied the effect of pneumonia on all-cause mortality after elderly hip fractures, which suggested an increase in mortality in pneumonia patients at 30 days to 1 year compared to non-pneumonia patients [26]. This could be attributed to the fact that the population used in their study is composed of patients over the age of 65, compared to the population in our study, which included patients from a wider range of age groups. The impact of COVID-19 pneumonia on overall mortality among hip fracture patients, which Fessler et al. studied, is another factor worth mentioning [27]. Several meta-analysis studies suggested a significant increase in mortality among patients with femoral or hip fractures who had a perioperative or concomitant COVID-19 infection [27–29]. In addition, in national research involving 3303 adults who underwent hip fracture surgery, the all-cause mortality for individuals who tested positive for COVID-19 was 27.0%, compared to 12.4% for those who tested negative for COVID-19 [30]. On the other hand, COVID-19 infection did not significantly modify 30-day and 6-month mortality, and in another study, they found no significant difference in 120-day mortality [31, 32]. This information is critical for orthopedic surgeons to consider when managing patients with femoral fractures and concomitant COVID-19 infection. This could have an effect on our mortality results because our population includes patients from before and after the pandemic.

In our study, older age was a significant predictor of postoperative pneumonia in patients after femoral fracture. Similar to our study results, in a study including 3147 patients, they found a postoperative pneumonia rate of 5.8%, and they found age to be an independent risk factor for postoperative pneumonia [18].n In a multicenter retrospective study, they found that older age was associated with a higher risk for aspiration pneumonia in patients with hip fractures [33]. Moreover, low hemoglobin levels on admission have been linked to increased severity and adverse outcomes in various infectious diseases [34, 35]. In the context of pneumonia, reduced hemoglobin levels may reflect compromised oxygen-carrying capacity, potentially exacerbating tissue hypoxia and impairing the immune responses that combat infection [35]. Furthermore, a previous study found that low hemoglobin levels upon admission were significantly associated with 6-month mortality in hip fracture patients [36]. In our study, lower hemoglobin levels were associated with prolonged hospital stays in the linear regression, but there was no significant difference in the hemoglobin level between the patients with postoperative pneumonia and those without.

The data in this paper ascertains that there are comorbidities more prevalent than others in those patients who developed postoperative pneumonia. These include hypertension, diabetes, and cardiovascular disease. However, only cardiovascular disease was significantly more prevalent in patients with prolonged LOS, regardless of the pneumonia diagnosis. While the specific comorbidities involved are not extensively discussed, a retrospective multi-center cohort study has found that broadly, the presence of preoperative comorbidities has been associated with a rise in LOS [7, 10]. In addition, evidence suggests an increased prevalence of comorbidities coinciding with the initial incidence of hip fractures. A study by Yu Jiang et al., which involved patients undergoing surgical treatment for hip fractures, found hypertension as the most prevalent comorbidity at 52.0% (67.8% in our study), followed by 23.6% with type 2 diabetes (49.4% in our study), coronary heart disease (20.9%), stroke (18.7%), and arrhythmia (11.2%) (combined cardiovascular disease prevalence in our study was 29.1%) [37].

Previous studies have linked hypertension to respiratory infections, specifically pneumonia [38]. Chronic hypertension commonly coexists with endothelial dysfunction, immunological dysregulation, and altered inflammatory responses, suggesting a complicated interaction [39, 40]. The higher prevalence (78.3%) of hypertension among patients with postoperative pneumonia in our study, warrants consideration. Also, hypertension has an established role in immune modulation and a potential impact on lung function [38–40]. Furthermore, similar to the previously mentioned effects of hypertension, diabetes also had several mechanisms that could increase their risk of infection, including increased altered immune cell function, bacterial proliferation, and changes in vascular permeability and endothelial cells, which was attributed to an increase in the incidence of postoperative pneumonia after an array of surgeries [41]. This element of immunosuppression may explain why diabetes was more prevalent (60.9%) in our cases of postoperative pneumonia. Nonetheless, its effects on increased LOS could be explained by an increase in other postoperative complications [42], which negatively impact surgical outcomes and require further interventions post-operatively. Moreover, it is well known that diabetes influences wound healing due to its deleterious effects on microcirculation and the metabolic pathway [43]. Cardiovascular diseases (CVD) were found to be a trigger for hemodynamic instability, which contributes to pulmonary congestion and edema that could result in an infection, possibly pneumonia [44]. Moreover, Lee et al. reported that for geriatric patients with femoral neck fractures undergoing hemiarthroplasty procedures, congestive heart failure doubled the chances of developing POP [45]. This further confirms our findings of increased POP and LOS in patients with cardiovascular diseases on univariate analysis. Preoperative cardiac evaluation guidelines set out by the American College of Cardiology/American Heart Association (ACC/AHA) categorize any orthopedic procedure, including femoral fracture repair, as “intermediate risk.” [46]. Specifically, heart failure has been previously found to increase LOS following hip fracture surgeries, which goes hand in hand with our findings [47].

A Danish study in 2019 confirmed our findings regarding postoperative pneumonia. It suggested that a delay of 12 h was associated with an increased risk of pneumonia in patients with no comorbidities, a delay of 24 h was associated with an increased risk of pneumonia in patients with a medium level of comorbidity, and a delay of 48 h was associated with an increased risk of reoperation due to infection in patients with a high level of comorbidity. In conclusion, a delay in surgery was associated with an increased risk of hospital-treated pneumonia and reoperations due to infection within 30 days of surgery [48]. Many articles have confirmed that a delay in surgery over 48 h is concurrent with worsening outcomes, hence increased LOS, reasoning that a delay in the performance of surgery is linked to major medical complications, minor medical complications, and pressure sores [49, 50]. Furthermore, prior research involving polytrauma patients has demonstrated that early stabilization of femur fractures is linked to a reduced risk of acute respiratory distress syndrome and mortality [51]. Interestingly, a retrospective review conducted in 2018 revealed that increasing time to surgery was associated with longer postoperative lengths of stay but not with adverse outcomes of surgery [52].

The retrospective study investigating the impact of pneumonia on the length of hospital stay and mortality in elderly femoral fracture patients exhibits several notable strengths. The study addressed a clinically significant issue by investigating the impact of pneumonia, specifically in femoral fracture patients. In addition, understanding the interplay between these two conditions can inform healthcare strategies and improve patient care. Furthermore, a larger sample increases the likelihood of detecting true associations, strengthens the study’s external validity, and utilizes multivariate analysis controlled for potential confounding variables. However, certain limitations warrant consideration. The study’s retrospective design is inherently limited by its reliance on existing medical records, which may lack some critical information. Moreover, conducting the study at a single healthcare center may limit the generalizability of the findings. Also, failure to account for nosocomial cases could underestimate the true impact of hospital-acquired infections on the studied outcomes. Future studies could benefit from incorporating ASA grades and utilizing the CURB-65 scoring system, which could potentially enrich the analysis and provide deeper insights into the prognostic factors influencing postoperative outcomes.

## Conclusion

In light of our findings, this study underscores the significant impact of postoperative pneumonia on the outcomes of patients undergoing femur fracture surgery. With a notable incidence of 10.8%, postoperative pneumonia was associated with older age, prolonged hospital stay, and intensive care unit (ICU) admissions, though it did not significantly affect mortality rates. In addition, prolonged hospital stay was associated with surgery not being performed within 48 h of admission, lower hemoglobin levels upon admission, and ICU admission. For clinicians, our study emphasizes the importance of early identification and management of risk factors for postoperative pneumonia. Implementing targeted interventions, such as preoperative optimization, timely surgical intervention, and enhanced postoperative care protocols, could mitigate the risk of developing pneumonia, improve overall outcomes, and lower the incidence of postoperative pneumonia in patients with femur fractures.

## Data Availability

The datasets generated and/or analysed during the current study are not publicly available due to the sake of patient privacy but are available from the corresponding author on reasonable request.
